# Type 3 Deiodinase: Role in Cancer Growth, Stemness, and Metabolism

**DOI:** 10.3389/fendo.2014.00215

**Published:** 2014-12-17

**Authors:** Domenico Ciavardelli, Maria Bellomo, Caterina Crescimanno, Veronica Vella

**Affiliations:** ^1^School of Human and Social Science, University “Kore” of Enna, Enna, Italy; ^2^Center of Excellence on Aging (CeS.I.), University “G. d’Annunzio” of Chieti-Pescara, Chieti, Italy; ^3^Department of Clinical and Molecular Bio-Medicine, Endocrinology Unit, University of Catania, Garibaldi-Nesima Medical Center, Catania, Italy

**Keywords:** deiodinase, cancer stem cells, stemness, Warburg effect, type 3 deiodinase

## Abstract

Deiodinases are selenoenzymes that catalyze thyroid hormones (THs) activation (type 1 and type 2, D1 and D2, respectively) or inactivation (type 3, D3). THs are essential for proper body development and cellular differentiation. Their intra- and extra-cellular concentrations are tightly regulated by deiodinases with a pre-receptorial control thus generating active or inactive form of THs. Changes in deiodinases expression are anatomically and temporally regulated and influence the downstream TH signaling. D3 overexpression is a feature of proliferative tissues such as embryo or cancer tissues. The enhanced TH degradation by D3 induces a local hypothyroidism, thus inhibiting THs transcriptional activity. Of note, overexpression of D3 is a feature of several highly proliferative cancers. In this paper, we review recent advances in the role of D3 in cancer growth, stemness, and metabolic phenotype. In particular, we focus on the main signaling pathways that result in the overexpression of D3 in cancer cells and are known to be relevant to cancer development, progression, and recurrence. We also discuss the potential role of D3 in cancer stem cells metabolic phenotype, an emerging topic in cancer research.

## Introduction

Thyroid hormones (THs) are iodinated compounds known to regulate a wide range of cellular activities through TH receptors (TRs). These are sequence-specific ligand-dependent transcription factors that trigger many of the THs downstream effects by activating or repressing target genes. TH functions are important for development, tissue differentiation, and maintenance of cell metabolic balance (1). Severe disruption of TH action during fetal and early neonatal development leads to permanent deficits (2).

Deiodination is a critical process by which the less active hormone thyroxin (T4) is converted into the more active form, triiodothyronine (T3). Deiodinases exert a major metabolic control of intracellular TH concentrations leading to a tissue-specific TH bioavailability. D2 and D3 are widely expressed but they are dynamically and tightly coordinated allowing cells to adapt their own TH activity. In contrast, the role of D1 is primarily to provide T3 for the circulation and it is highly expressed in the liver, kidney, and thyroid (3).

D3 is the physiological inactivator of TH that acts by deiodinating the inner ring of T3 and T4 hormones to give T2 and reverse T3, respectively. So far, this enzyme controls local TH homeostasis and protects tissues from TH excess. Local deiodination represents an example of cell-autonomous, pre-receptoral control of TH action showing that TH signaling can be different in tissues even in the presence of the same serum hormone concentration (4, 5).

D3 plays a major role in lowering serum TH concentrations during development. Furthermore, the expression levels and activity of D3 significantly increase in the embryo tissues such as liver, cerebral cortex, gonads, umbilical arteries and vein, lung, heart, intestine, and skin (6) compared to the adult tissues. D3 is also highly expressed in the human placenta where it has to reduce fetal tissues exposure to TH. In contrast, D3 activity has been identified in a limited number of postnatal tissues as brain, skin, and pregnant uterus.

Mouse model of D3 deficiency (D3KO) has been pivotal in current understanding of the functional role of D3. D3KO mice showed impaired fertility, significant perinatal mortality, and growth impairment. In addition, the hypothalamic–pituitary–thyroid (HPT) axis development was altered probably for the overexposure to excessive levels of THs (7). Notably, these abnormalities resemble those observed in children exposed to high levels of THs because of their mothers’ hyperthyroidism during pregnancy (8, 9).

Overall, these findings point out the key role of D3 in cell proliferation and differentiation in normal and pathological conditions. In particular, several studies, recently reviewed by Casula and Bianco (10), demonstrated that the local control of THs signaling provided by the regulation of D3 activity is strictly associated to cancer development, progression, and recurrence.

This review focuses on the most recent progresses achieved in this field, particularly the physiological function and significance of D3 in the stemness and metabolic features of cancer cells.

## Type 3 Deiodinase: Role and Functions in Normal and Cancer Tissues

D3 has been defined as an “oncofetal” protein because highly expressed during embryonic development and cancer. Beyond its considerable role in protecting tissues from excessive TH levels during embryogenesis, D3 exerts a relevant function also in adult life. Several studies revealed that signals such as hypoxia or ischemia induce D3 re-expression in pathological conditions as cancer, cardiac hypertrophy, myocardial infarction, chronic inflammation, and critical illness, indicating that cell-specific TH inactivation is critical in these conditions (11, 12). In adult life, D3 expression ceases and switches on upon tumoral transformation, while it remains silent in the normal counterpart tissues. D3 reactivation in adult tissues has been correlated with hyperproliferative conditions and has been found in human solid tumors. This finding suggests a link between deiodinase-mediated TH metabolism and carcinogenesis. Indeed, D3-mediated local hypothyroidism promotes cellular proliferation by regulating the nuclear T3 availability.

It has been reported that immortalized cell lines derived from different tumors such as basal-cell carcinoma (BCC), hemangiomas, hepatocarcinomas, breast cancer (MCF-7 cells), colon adenocarcinoma (Caco2, SW280, and HCT116 cells), thyroid cancer, endometrium cancer (ECC-1 cells), and neuroblastoma (SH-SY5Y cells) express elevated D3 levels (13–16).

D3 overexpression has been further verified in many human cancer tissues. Vascular tumors (hepatic hemangiomas) show high D3 activity resulting in an accelerated rate of THs degradation leading to a clinically relevant hypothyroidism named “consumptive hypothyroidism” (17, 18). Elevated levels of D3 decrease nuclear TH availability in BCC cells compared to normal keratinocytes, probably enhancing the proliferation rate of these cells. D3 is also up-regulated in papillary thyroid cancer (PTC) with tumors harboring BRAF^V600E^ mutation having the highest levels of D3 activity. In those cancers, increased D3 activity positively correlates with tumor size and disease spread (19). Since thyroid cancer is characterized by altered tumor suppressor p53 family members expression (20–23), it should be interesting to analyze if there is any correlation with deiodinases deregulation. We could also hypothesize that restoration of tumor suppressor activity (24) could modify D3 expression.

In benign adenomas and in colon carcinomas, D3 expression is significantly higher than in normal tissues but negatively correlated with the histologic grade of the lesions suggesting that D3 could be a marker of the early stages of tumorigenesis (16).

## Molecular Mechanisms of D3 Regulation in Cellular Pathways Involved in Stemness

Several signaling molecules involved in the control of cell proliferation such as hormones and growth factors stimulate D3 expression. Estrogens and progesterone independently increase D3 expression in the uterus (6). Furthermore, D3 expression is strongly induced by epidermal (EGF) and fibroblast growth factors (FGF) (25). In *in vitro* experiments on astrocytes and preadipocytes, serum and phorbol esters induce D3 expression (26), a stimulatory effect that appears to be mediated, at least in part, by the activation of the MEK/ERK signaling cascade (27). Notably, D3 expression is under the control of signaling pathways linked to stemness and involving sonic hedgehog-glioma associated oncogene 2 (Shh-Gli2), Wnt/β-catenin, tumor growth factor-β (TGF-β), and hypoxia-inducible factor-1α (HIF-1α) (16, 28–31) (Table 1).

**Table 1:** **Hormones and intracellular pathways inducing D3 overexpression and subsequently local hypothyroidism**.

	D3 inducers	Involvement in cancer cell stemness
Estrogen	Bates et al. (6)	
Progesteron	Bates et al. (6)	
EGF	Hernandez et al. (25)	
FGF	Hernandez et al. (25)	
Serum	Courtin et al. (26)	
Phorbol compounds	Courtin et al. (26)	
Shh-Gli2	Dentice et al. (28)	Ahn et al. (37); Bhardwaj et al. (35); Goodrich et al. (41); Paladini et al. (77)
Wnt/β-catenin	Dentice et al. (16)	Sirakov et al. (48)
TGF-β	Huang et al. (29)	Massague et al. (50)
HIF-1α	Simonides et al. (31)	Keith et al. (57); Li et al. (58); Wang et al. (59); Ciavardelli et al. (62); Morfouace et al. (65)

*Pathways significantly involved in cancer stemness are also shown*.

### The Hedgehog cascade

Vertebrates possess three hedgehog (Hh) proteins, Sonic hedgehog (Shh), Indian hedgehog (Ihh), and Desert hedgehog (Dhh), all of which bind to the receptor patched homolog-1 (PTCH-1). SHH is the best-studied ligand of the vertebrate pathway that signals in an autocrine or paracrine fashion (32).

In the absence of the SHH ligand, PTCH-1 inhibits a downstream protein in this pathway named Smoothened (SMO). PTCH-1 removes oxysterols from SMO reducing its activity (33). Upon binding of an Hh protein or a mutation of PTCH, SMO oxysterols increase. This accumulation allows SMO to activate the zinc-finger transcription factors Gli. The sequence of the molecular events that connect SMO to Gli is not completely understood. Activated Gli accumulates in the nucleus and controls the transcription of hedgehog target genes that play important roles in a wide variety of developmental processes (34). Indeed, disruption of hedgehog signaling during embryonic development, through either deleterious mutation or consumption of teratogens by the gestating mother, can cause severe developmental abnormalities. However, Hedgehog signaling has an important role even in the adult life. SHH has been shown to promote adult stem cells proliferation in many tissues including primitive hematopoietic cells (35), mammary (36), and neural stem cells (37).

Of note, abnormal activation of this pathway probably leads to transformation of adult stem cells into cancer stem cells (CSCs). In fact, it has been implicated in the development of cancers in brain, lung, mammary gland, prostate, and skin (38, 39). BCC, the most common cancer of hair follicle-derived tumors in humans, has the closest association with the hedgehog signaling (40). Furthermore, loss-of-function mutations in PTCH (41) and activating mutations in SMO have been identified in patients with this disease (40).

There are several evidences that D3 is under the control of the Shh–Gli pathway. The activation of this pathway results in D3 overexpression in mouse and human BCC (28). By directly inducing D3 in keratinocytes, Shh causes a hypothyroid state at intracellular level resulting in a higher proliferative rate. Furthermore, during development another member of the Hh family, Ihh, reduces intracellular T3 availability by inducing D2 ubiquitination and degradation (42). However, the mechanisms that link the regulation of T3, via deiodinase action, to the Hh pathway are still unclear. In most adult tissues, Gli proteins have a short half-life and are poorly detectable, because of their rapidly degradation by proteasomes. Conversely, Hh-induced BCC tumorigenesis causes Gli protein stabilization and accumulation (43). It has been shown that D3 expression in BCC cells determines a proliferative advantage for the tumor and that, conversely, D3 depletion interferes with tumorigenesis, enhancing the apoptotic process. By promoting Gli degradation, T3 blocks its transcriptional activity. Moreover, T3 treatment or D3 depletion reduce tumor growth in a genetically modified mouse model of skin tumorigenesis. By this mechanism, Shh induces TH signaling attenuation contributing to epidermal tumorigenesis *in vivo* by favoring cell proliferation and bypassing T3-induced growth arrest and cell differentiation.

### The Wnt/β-catenin signaling

Wnt signaling is activated when specific ligands bind to frizzled and low-density lipoprotein receptor-related protein (LRP) receptors, allowing stabilization and nuclear translocation of β-catenin (44). The interaction between E-cadherin and the cytoskeleton with β-catenin participates in the regulation of actin filament assembly and cell adhesion (45). A complex of several protein kinases that promote β-catenin degradation regulates its cellular localization. In the presence of Wnt ligands, this complex is blocked so that β-catenin accumulates and translocates to the nucleus modulating Wnt target genes expression (44).

The *Wnt* genes encode a large family of cysteine-rich secreted polypeptides that mediate different signaling processes. Deregulation of Wnt signaling causes developmental defects and tumorigenesis (46). Indeed, the Wnt/β-catenin signaling pathway is deregulated in many colon tumors (16). As Wnt/β-catenin, also THs are involved in the control of intestinal development and proliferation, in fact in amphibian metamorphosis, they control gastrointestinal tract remodeling (47).

It has been recently shown that Wnt/β-catenin pathway directly affects TH signaling by a dual convergent mechanism, which modulates the activity of the deiodinase enzymes in colon cancer cells (16). Specifically, the TH activating D2 enzyme is down-regulated by β-catenin while D3 is over-expressed. Similarly to colon cancer, it should be postulated that deregulated Wnt pathway could induce D3 expression in other tumors.

In the intestinal epithelium, several signaling pathways including Wnt and TH are fundamental during development. In the adult life, they participate to maintain epithelial homeostasis and renewal through the intestinal stem cells localized in the crypt base. It has been demonstrated that, in the intestinal epithelium, precursor cells interact with the Wnt pathway molecules through their TRs. This interaction controls crypt proliferation in physio-pathological conditions (48). Interestingly, the same pathways are involved in retina development and may control the balance of adult stem/progenitor cells. Therefore, the cross-regulation between TRs and the Wnt pathway could be important in the adult stem cells biology, as demonstrated in both retina and intestinal systems.

### The TGF-β signaling

TGF-β family members control cell differentiation, migration, growth, and neoplastic transformation (49, 50). The classic TGF-β signaling cascade is a linear pathway involving two cell surface receptor kinases that, when activated, phosphorylate one or more Smad proteins, causing them to enter the nucleus and activate target gene transcription (50–52). Regulation by TGF-β can synergize with other ligands such as TPA, EGF, and FGF, known to signal through trans-membrane receptor tyrosine kinases.

Of note, TGF-β regulates THs signaling. In fact, D3 mRNA is transcriptionally stimulated by TGF-β in different human cell types including fetal and adult fibroblasts, fetal epithelia, skeletal muscle myoblasts, uterine endometrium, hemangioma cells, and gliomas (29). As described for other pathways, local hypothyroidism induced by TGF-β could facilitate the expression of oncofetal genes and inhibit the differentiation effects of TH or promote cell survival in pathological conditions as ischemia or inflammation reducing their metabolic requirements.

### The HIF-1 signaling

Several tissues that express D3 are known to be hypoxic, including normal tissues of the human fetus (53) and ischemic tissues of critically ill patients (54). Local TH inactivation by D3 may be an important component of the tissue response to different hypoxic–ischemic injuries.

Although the regulation of D3 expression by oxygen availability is cell-type specific, hypoxia increases D3 mRNA and activity in different cell types. HIF-1α is the transcriptional mediator of the cellular response to low oxygen and hypoxia mimetics such as desferrioxamine (DFO) and CoCl2, which promote HIF-1α stabilization and accumulation, also induce D3. In fact, strong evidences show that D3 is a direct HIF-1 target gene. Increased D3 activity inhibits T3-stimulated metabolic rate in cells *in vitro* and reduces specific T3 activities *in vivo* (31).

Because THs are potent stimulators of metabolic rate and oxygen consumption, the regulation of local T3 action in hypoxic tissues has important physiological consequences. Many HIF-1 target genes are known to promote the survival of hypoxic cells (31). Noteworthy, endogenous D3 activity is a potent inhibitor of T3-dependent oxygen consumption. This suggests a mechanism of metabolic regulation during certain hypoxic–ischemic injuries in which HIF-1-induced D3 promotes the viability of hypoxic tissues by reducing T3-stimulated energy expenditure (31). The T3-dependent reduction of oxygen consumption supports the idea that D3 reactivation in certain conditions may be considered advantageous for the cell. Furthermore, the ability of D3 to decrease the metabolic rate and the oxygen consumption is consistent with the function of many other HIF-1 target genes (53). Of note, hypoxia is a common feature of advanced solid tumors and has been associated with poor therapeutic response and increased risk of recurrence (55, 56). HIF has been shown to activate specific signaling pathways that control stem cell self renewal and multipotency (57–59). However, several HIF-1 target genes, such as the key enzymes of the first phase of cell respiration, are over-expressed in cancer cells also upon normoxic conditions, resulting in increased fermentative glycolysis. This phenomenon has been termed “the Warburg effect” and has been associated to tumor progression and aggressiveness (60). This may indicate that overexpression of D3 in cancer cells is not merely a response to hypoxia but may significantly contribute to the complex mechanisms that proliferating cancer cells set in motion also upon normoxic condition in order to decrease the energy substrate degradation, thus supporting the diversion of metabolites into cellular biosynthetic pathways.

## Type 3 Deiodinase and Warburg Effect

The study of cancer metabolism has recently gained great attention as pharmacological targeting of specific metabolic pathways is a promising therapeutic approach and cancer cell proliferation is strictly related to significant alterations of cellular metabolism (61).

As discussed above, cancer cells are characterized by increased aerobic glycolysis and lactic acid production upon normoxic condition compared to normal cells. Recent studies indicate that this metabolic phenotype is strictly associated to the stemness of cancer cells (62–64). It is worthy of note that this metabotype is linked to the increased chemoresistance of CSCs compared to more differentiated tumor cells. In fact, pharmacological interventions aimed to differentiate CSCs result in a metabolic shift from aerobic glycolysis toward mitochondrial oxidative phosphorylation and increase cell death (65). As CSCs are involved in tumor resistance, metastasis formation and reliance, the effective elimination of CSCs is critical for successful therapeutic outcomes (66, 67). Although it is well known that D3 promotes the proliferation of malignant cells and tumor growth, it is still unclear whether D3-induced local hypothyroidism may have a role in determining the metabolic phenotype and aggressiveness of CSCs. In fact, data on D3 activity and expression in CSCs are currently unavailable. On the other hand, it is known that thyroid hormones (THs) T3 and T4 modulates Warburg phenotype in breast-cancer cells and treatment with T3 decreases the tumor chemoresistance *in vitro* inhibiting aerobic glycolysis and increasing mitochondrial activity and oxygen consumption (68). This finding suggests that the increase of T4 and T3 deiodination by D3 could be associated to more aggressive phenotypes.

**Figure 1:**
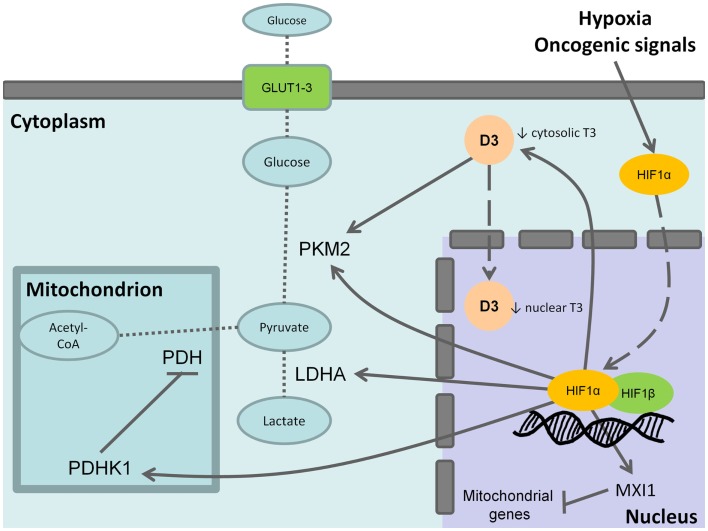
**Effects of type 3 deiodinase expression on Warburg phenotype**. Hypoxia or oncogenic signals inhibit HIF-1a degradation and stabilize the nuclear association between HIF-1a and HIF-1b resulting in the transactivation of HIF-1 target genes. The activation of the M2 isoform of pyruvate kinase (PKM2), lactate dehydrogenase A (LDHA), and of the pyruvate dehydrogenase kinase 1 (PDHK1) that, in turn, inhibits the mitochondrial pyruvate dehydrogenase (PDH) shunts cell metabolism from the mitochondrial respiration toward the fermentative glycolysis. Furthermore, the induction of max interactor 1 (MXI1), a transcriptional target of HIF-1 complex, inhibits mitochondrial biogenesis through the downexpression of nuclearly encoded mitochondrial genes. The coexpression of type 3 deiodinase (D3) decreases cytosolic triiodothyronine (T3) levels resulting in the activation of PKM2. It is also possible that D3 translocates from cytoplasm to the cell nucleus mediating nuclear thyroid hormone inactivation and local hypothyroidism. Bold arrows indicate activation, whereas the blunted lines indicate inhibition. Dashed arrows indicate protein translocation between cellular compartments. Dotted lines indicate the pathway reactions.

As discussed above, D3 is involved in the adaptative response to hypoxia in cells and is trascriptionally induced by HIF-1α (31) as well as other molecular mediators of the Warburg effect such as hexokinase, pyruvate kinase (PK), and lactate deydrogenase (Figure 1). Notably, the embryonic and low activity isoform of PK, PKM2, is enriched in CSCs and promotes the aerobic glycolysis thus contributing to anabolic metabolism. T3 has been reported as an allosteric inhibitor of PKM2 *in vitro* (69, 70). Therefore, the overexpression of D3 and the resulting decrease of cytosolic T3 increase PKM2 activity stimulating the aerobic glycolysis (Figure 1). Furthermore, in neurons, hypoxia induces heat-shock protein-40 (Hsp-40)-mediated nuclear import of D3 that facilitate THs inactivation resulting in nuclear hypothyroidism and reducing cellular metabolism and oxygen consumption (71). Although this mechanism has not been yet reported in cancer cells, it is worthy of note that Hsp-40, also known as DNAJB1, and the other members of DNAJ family have been found to be involved in the regulation of cancer cells and CSCs (72). The function of Hsp-40 is still controversial because this chaperone protein may interact with PKM2 inducing its degradation and impairing tumor cell proliferation (73). However, some CSCs isolated from specific solid tumors display increased levels of Hsp-40 (74). Therefore, it is intriguing to speculate that D3 could contribute to the glycolytic phenotype of CSCs and therefore provide an additional target to counteract CSCs metabolism.

## Conclusion

At cellular level, TH signaling can be regulated by deiodinases in a time- and tissue-specific fashion independently on plasma thyroid hormone levels (11). Although it is not clear why deiodinases expression is altered in cancer tissues, this could be used as a marker of the disease. Nevertheless, changing in TH signaling through deiodinase activation could play a role in cell proliferation or viability. Since attenuation of TH signaling is part of a widespread neoplastic program, D3 expression may therefore be one of the mechanisms used by cancer cells to get rid of the THs differentiation action.

An increase in TH signaling accelerates the mitochondrial oxidation of energy substrates such as glucose and fatty acids in most cells types (75). As cancer cells and CSCs are known to mainly rely on aerobic glycolysis (76) that is stimulated by cell hypothyroidism, it is conceivable that deiodinases modulation could be a potential therapeutic target for cancers that depend on high metabolic rate and do not show deiodinases deregulation.

## Conflict of Interest Statement

The authors declare that the research was conducted in the absence of any commercial or financial relationships that could be construed as a potential conflict of interest.
